# A Rare Case of Multiple Myeloma Identified Following the Diagnosis of Amyloidosis of the Tongue

**DOI:** 10.1155/2024/8836103

**Published:** 2024-10-28

**Authors:** Hideka Kanemoto, Kyoichi Obata, Koichi Kadoya, Kisho Ono, Hotaka Kawai, Yuki Kunisada, Mayumi Yao, Soichiro Ibaragi

**Affiliations:** ^1^Department of Oral and Maxillofacial Surgery, Okayama University Graduate School of Medicine, Dentistry and Pharmaceutical Sciences, 2-5-1 Shikata, Okayama 700-8525, Japan; ^2^Department of Oral and Maxillofacial Surgery, Kochi Health Sciences Center, 2125-1 Ike, Kochi 781-8555, Japan; ^3^Department of Oral and Maxillofacial Surgery, Tsuyama Chuo Hospital, 1756 Kawasaki, Tsuyama, Okayama 708-0841, Japan; ^4^Department of Oral Pathology and Medicine, Okayama University Graduate School of Medicine, Dentistry and Pharmaceutical Sciences, 2-5-1 Shikata, Okayama 700-8525, Japan

## Abstract

Amyloidosis is a disease in which amyloid protein is deposited in organs and tissues, resulting in functional impairment. Amyloidosis occurs in 12%–30% of patients with multiple myeloma, but in rare cases, amyloidosis may precede the diagnosis of multiple myeloma. Our patient was a 76-year-old Japanese male on dialysis. Multiple nodules accompanied by ulcers were observed on his tongue. He had no subjective symptoms or clinical findings associated with multiple myeloma. The histopathological findings suggested amyloidosis. We suspected both systemic and localized amyloidosis and performed a comprehensive systemic examination. Since the patient had been on dialysis for only a short period of time (~3 months), dialysis-related amyloidosis was ruled out. After blood and urine tests, a diagnosis of multiple myeloma was made. Chemotherapy treatment was started, but the patient's multiple myeloma could not be suppressed and the tongue amyloidosis worsened, leading to his death 2 years and 2 months after the initial diagnosis.

## 1. Introduction

Amyloidosis is a refractory metabolic disease of unknown cause in which amyloid proteins deposit in various organs and tissues throughout the body, leading to functional impairments. The clinical symptoms of amyloidosis are diverse and depend on the organ dysfunction. Amyloid proteins are classified essentially based on their precursor proteins, which form the amyloid. Forty-two types of amyloid precursor substances have been identified in humans to date [1, 2]. Although there are differences in the formation and deposition mechanisms of amyloids depending on the precursor substances, amyloidosis typically develops when these precursor substances are produced; the substances then undergo fragmentation and polymerization and form *β*-sheet aggregates [3–5].

Amyloidosis can be classified into systemic and localized types. In systemic amyloidosis, amyloids deposit in multiple organs such as the heart, kidneys, and nervous system, leading to dysfunction. In localized amyloidosis, amyloid deposits are confined to a single organ [1]. The most common site of amyloidosis is the tongue [6], presenting with irreversible multiple soft, dark-purple tumors and macroglossia, leading to speech and swallowing difficulties. In severe cases, amyloidosis can cause respiratory distress [7–10]. Although surgical removal of the tumor or reduction surgery of the tongue is recommended for localized amyloidosis and for patients with functional impairments, systemic amyloidosis requires treatment of the underlying disease, necessitating the exclusion of systemic amyloidosis when amyloidosis is discovered in the oral region [8].

Multiple myeloma (MM) is characterized by a monoclonal proliferation of plasma cells and an increase in the level of monoclonal immunoglobulin (M protein) in the serum and urine. There are four main sets of symptoms of MM, referred to as CRAB (calcium elevation, renal failure [kidney failure], anemia, bone disease): (i) malaise, tiredness, and black mouth due to hypercalcemia; (ii) swelling and oliguria due to renal involvement; (iii) palpitations and dizziness due to anemia; and (iv) bone pain and pathological fractures due to bone lesions. However, in many cases, there are no symptoms in the early stages of the disease, and the disease is discovered by chance during a physical examination or test performed for other diseases [11, 12].

MM is a malignancy that is very difficult to treat radically, despite the discovery of many new treatments, with a 5-year survival rate of approximately 50%–60%. Thirty percent of MM patients have amyloid deposits in the bone marrow without organ damage. However, 12%–15% of patients develop amyloidosis due to amyloid deposits in organs as MM progresses [13, 14]. Amyloidosis is usually discovered during the treatment of patients with MM, but in rare cases, MM may be detected during the search for the cause of amyloidosis. Our literature review identified seven cases in which oral symptoms were observed before a diagnosis of MM was made [15–20].

We present the case details of a patient whose amyloidosis was identified in the tongue. A comprehensive systemic examination subsequently led to a diagnosis of MM.

## 2. Case Report

**Figure 1:**
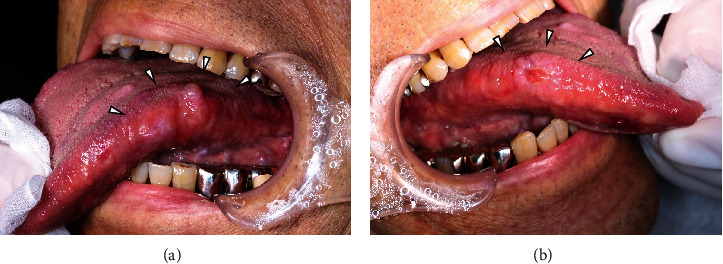
Intraoral photographs at initial examination (a) on the left and (b) on the right. The left and right lingual margins have yellowish-pink nodules (white arrowheads).

A 76-year-old Japanese man was referred in January 2018 to the Department of Oral Surgery, Tsuyama Chuo Hospital, from his general dental clinic for the investigation of multiple masses on the bilateral border of his tongue. He had been treated for polycystic kidney disease since 1998 and had been undergoing dialysis since November 2017 due to decreasing renal function. An oral examination revealed multiple painless, soft, small nodules extending from the bilateral border to the dorsum of the tongue. These small nodules were yellowish-pink with whitish changes on the surface. The border of the right tongue revealed ulceration (Figure 1). Oral intake was possible, and mild speech impairment was noted. There were no remarkable clinical findings and no patient symptoms, such as CRAB, suggestive of MM.

**Figure 2:**
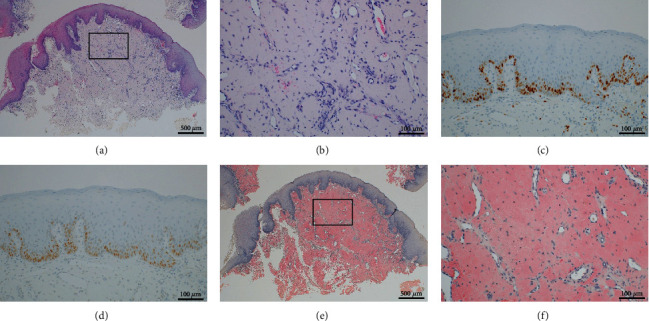
No epithelial cell atypia was observed in (a) (hematoxylin and eosin [HE] stain, ×4), while amyloid deposition is evident beneath the epithelium in (b) (HE stain, ×20). The immunohistochemical staining results using Ki-67 and p53 indicate the absence of malignant findings in (c) and (d) ((c) Ki-67 and (d) p53, ×20). Extensive amyloid deposition beneath the epithelium was observed, stained orange by Congo red staining in (e) and (f).

A physician who had performed the patient's dialysis indicated that there were no abnormalities of note other than decreased renal function. With the clinical diagnosis of multiple tongue tumors, the patient underwent a biopsy under local anesthesia for a histological examination which revealed extensive eosinophilic amorphous homogeneous deposits in the subepithelial connective tissue. There was no cellular atypia in any of the cells in the biopsy material. Direct fast scarlet (DFS) staining and Congo red staining showed orange staining in the same area. Based on these features, a diagnosis of tongue amyloidosis was made (Figure 2).

In light of the patient's multiple episodes of amyloidosis, we suspected systemic amyloidosis rather than focal amyloidosis. Since the patient had been on dialysis for only 3 months, dialysis-related amyloidosis (DRA) was ruled out. He was referred to a nearby hematology/oncology department for a thorough examination, and the blood tests conducted there revealed a normal IgG level (888 mg/dL, reference range 861–1747 mg/dL) but low IgA (85 mg/dL, ref. 93–393 mg/dL) and IgM (21 mg/dL, ref. 33–183 mg/dL) levels. A urinalysis was positive for Bence–Jones protein. This led to the diagnosis of AL amyloidosis of the tongue and MM.

After consultation with a local hematologist/oncologist, a treatment plan was selected: chemotherapy would be started immediately and the tongue tumor would be resected in order to improve the patient's masticatory and swallowing functions if problems occurred. Treatment with IRD (ixazomib, lenalidomide, dexamethasone) was initiated in June 2018, but no change in disease status was observed. Treatment with MPB (melphalan, prednisolone, bortezomib) was started in November 2018, but due to adverse events, IRD therapy was resumed in January 2019.

However, despite continued chemotherapy, the patient's response was poor, and his condition deteriorated. The extent and size of the tongue amyloidosis worsened accordingly, and oral intake became difficult. A partial tongue resection was considered but was not indicated due to the patient's poor general condition. In March 2020 (2 years and 2 months after the initial diagnosis), the patient died of aspiration pneumonia.

## 3. Discussion

The most well-known classification of amyloidosis is the four-category classification proposed by Reimann, Koucky, and Eklund in 1935 [21]: the first category is primary amyloidosis, in which fibrous aggregates of monoclonal immunoglobulin light chains are deposited in vital organs such as the liver, heart, kidneys, and spleen. The second category is secondary amyloidosis, a serious complication of a primary disease such as MM, rheumatoid arthritis, chronic abscess, or Hodgkin's lymphoma. The third category is hereditary or familial amyloidosis, caused by inherited genetic mutations resulting in the lifelong production of abnormal proteins. The fourth category is focal amyloidosis, which is not associated with MM or systemic amyloidosis and does not progress to systemic amyloidosis [6, 21–23].

More recently, however, amyloidosis is commonly classified based on the causative amyloid proteins. Forty-two types of amyloid precursor proteins have been identified; of these, 14 types appear only in systemic amyloidosis, 24 appear only in localized amyloidosis, and four appear in both systemic and localized forms. The main subtypes of systemic amyloidosis are AL amyloidosis and ATTR amyloidosis (with amyloid deposits made up of transthyretin [TTR]). The incidence of AL amyloidosis is 10 cases per million people per year [1, 2, 24, 25]. At diagnosis, approximately 70% of AL amyloidosis patients suffer from severe organ damage. The kidneys and heart are particularly susceptible, followed by the liver, gastrointestinal tract, and autonomic nervous system. Due to the poor prognosis associated with kidney and heart damage, patients with unexplained nephrotic syndrome, renal failure, or congestive heart failure should be carefully evaluated for the presence of AL amyloidosis [26, 27]. Although a generalized examination was performed for the present patient, it revealed no evidence of amyloidosis other than on the tongue.

Amyloidosis caused by *β*2-microglobulin (*β*2M) is also referred to as DRA. *β*2M is normally filtered by the renal glomeruli and then largely reabsorbed and broken down in the proximal tubules. In individuals who have end-stage renal disease (in which kidney function is compromised), *β*2M is not adequately filtered by the kidneys, leading to increased concentrations in the blood. Because the molecular weight of *β*2M is only 11.8 kDa, it cannot be completely removed by dialysis. Among individuals who are undergoing long-term dialysis, this results in further increases in the blood level of *β*2M and its accumulation in the body, leading to the onset of the disease. Thus, although DRA does not occur in the short term, ~20% of patients undergoing dialysis develop DRA within 2–4 years of starting dialysis, and by 13 years, 100% of patients are affected [26, 28]. Our patient had been on dialysis for only 3 months, and dialysis amyloidosis was thus ruled out.

Both systemic amyloidosis and local amyloidosis are rare in the oral region. Reports of tongue amyloidosis have indicated that it is often systemic AL amyloidosis associated with plasma cell dyscrasia or MM. According to a systematic review of amyloidosis in the oral cavity by Pontes et al., AL amyloidosis was the most common, followed by DRA. The related diseases most frequently associated were MM, followed by renal failure [7]. However, many of these cases were secondary instances in which amyloidosis developed after the onset of MM, due to the deposition of amyloid derived from the light chains of M proteins [5]. Cases in which MM was discovered after a full-body examination following the diagnosis of amyloidosis are extremely rare [15, 16]. It may be that patients have simply been unaware that they had MM until symptoms of AL amyloidosis appeared.

For the present patient, we explored the possibility that he had asymptomatic MM, so-called monoclonal gammopathy of undetermined significance (MGUS), or smoldering MM (SMM). These diseases have no symptoms (including CRAB symptoms) and no organ damage, but they produce abnormal results on various blood and urine tests [29]. Xu et al. reported that amyloidosis is a poor prognostic factor for SMM, and the present patient's case may have been an instance of SMM [13]. Amyloidosis in the oral region of a patient with SMM has not been reported, to the best of our knowledge. However, in our patient's case, a definitive diagnosis as to whether he had SMM could not be obtained because of the abnormal blood and urine laboratory values since the patient was on dialysis. There have been reports of a late onset of MM during the treatment of patients with amyloidosis without concurrent MGUS or SMM [30, 31].

Regardless of the clinical course, the prognosis for patients with both MM and AL amyloidosis is very poor. A 49.5% mortality rate for patients with MM without AL amyloidosis (with a survival time of 42 months) was described by Xu et al., whereas for patients with both MM and AL amyloidosis, the mortality rate is 63.3% and the average survival time is 25.0 months [13]. Vela-Ojeda et al. stated that AL amyloidosis is a high-risk factor that worsens the prognosis of MM [32].

Our patient's case highlights the possibility that oral amyloidosis may be AL amyloidosis associated with MM, which has a very poor prognosis. When clinicians encounter a patient with oral amyloidosis, it is important to take prompt and appropriate action, with a thorough investigation of the patient's history and an extensive systemic examination.

## 4. Conclusion

We have provided the details of a rare case of MM diagnosed based on the results of a systemic examination after a prior diagnosis of lingual amyloidosis. When oral amyloidosis is encountered, it is important to promptly consult a specialist department to determine the presence/absence of MM.

## Data Availability

All data related to the present case are included in this published article.
